# Change in glycated haemoglobin in adults with type 2 diabetes on basal‐bolus insulin regimens following commencement of Freestyle Libre use

**DOI:** 10.1111/dom.16003

**Published:** 2024-10-10

**Authors:** Anna R. Dover, Rohana J. Wright, Shareen Forbes, Mark W. J. Strachan, Roland H. Stimson, Fraser W. Gibb

**Affiliations:** ^1^ Edinburgh Centre for Endocrinology & Diabetes Edinburgh UK; ^2^ University/BHF Centre for Cardiovascular Science, University of Edinburgh Edinburgh UK

**Keywords:** continuous glucose monitoring (CGM), glycaemic control, insulin therapy, type 2 diabetes

## 1 BACKGROUND

Most of the evidence supporting the efficacy of the Freestyle Libre system has accrued in people with type 1 diabetes. A randomized controlled trial of Freestyle Libre use in people with type 2 diabetes on intensive insulin therapy did not demonstrate any significant change in glycated haemoglobin (HbA1c), although hypoglycaemia was significantly reduced.^1^ More recently, real‐world evidence has suggested that Freestyle Libre use is associated with clinically relevant reductions in HbA1c in those with elevated HbA1c at baseline using intensive insulin therapy.^2^, ^3^ A number of studies have also suggested a potential benefit of continuous glucose monitoring in non‐insulin‐treated type 2 diabetes.^4^ Follow‐up duration in the existing literature is relatively short (up to 6 months), therefore, we sought to assess longer term outcomes in people with type 2 diabetes.

## 2 METHODS

This was an observational study of adults with type 2 diabetes attending a single diabetes centre in Scotland. As a service evaluation of routinely collected data, this project did not require ethical approval. All adults with type 2 diabetes on multiple daily insulin injection regimens were eligible for a funded Freestyle Libre device in our centre. Individuals were included if: (1) they were established on a basal‐bolus insulin regimen for >12 months and (2) they had HbA1c measured in the 12 months preceding and the 3–12 months following commencement. A total of 770 people with type 2 diabetes, on basal‐bolus regimens, were identified as Freestyle Libre users, however, paired HbA1c data were available in 304 (40%). For logistic regression analysis, a large cohort of people with type 1 diabetes was added (*n* = 987) to determine whether diabetes type predicted glycaemic response. Results are presented as median (interquartile range [IQR]). Paired data were compared using Wilcoxon signed‐rank tests and unpaired data using Wilcoxon rank‐sum test. Correlations were assessed by use of Spearman's correlation coefficient. Categorical data were compared using chi‐squared tests. *p* values <0.05 were taken to indicate statistical significance. Statistical analyses were performed using R Studio (version 2023.12.1).

## 3 RESULTS

In this study cohort, 57% of the participants (173/304) were male. The median (IQR) age was 61 (52–69) years and diabetes duration was 18 (12–23) years. The median (IQR) HbA1c at baseline was 73 (60–87) mmol/mol and body mass index (BMI) was 31.6 (27.6–36.3) kg/m^2^. The proportion of the cohort who initially commenced using the first‐generation Freestyle Libre was 43%, with the remainder commencing use of Freestyle Libre 2; this was not associated with any difference in outcomes.

**FIGURE 1:**
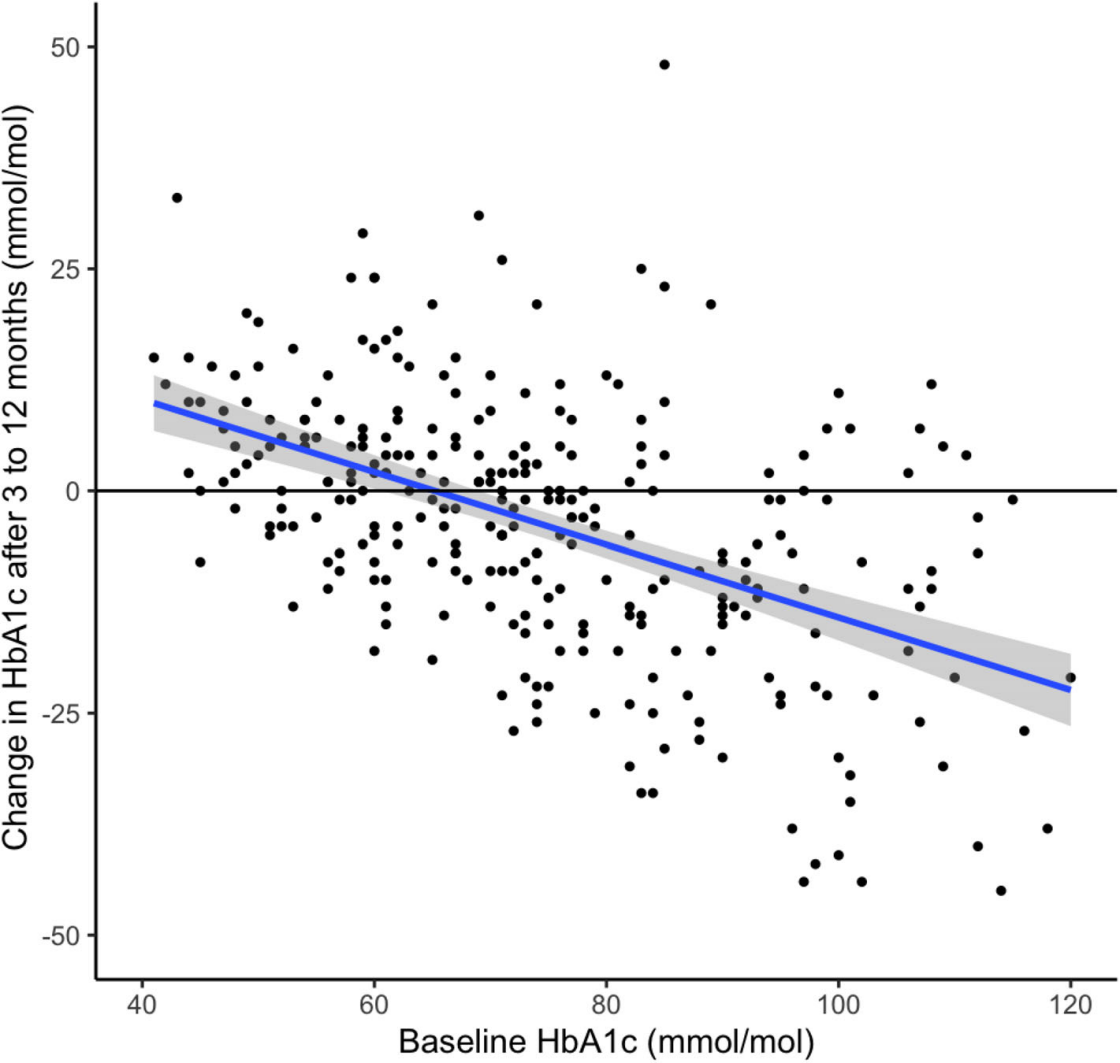
Relationship between baseline glycated haemoglobin (HbA1c) and subsequent change in HbA1c at 3–12 months (*r* = −0.514, *p* < 0.001).

Changes in HbA1c for the total cohort and stratified by baseline HbA1c are presented in Table 1. Overall, a small reduction in HbA1c was noted across 3 years of follow‐up, with a larger sustained reduction in HbA1c observed in those with highest baseline HbA1c and a small rise in HbA1c in those with the lowest HbA1c at baseline. Higher HbA1c at baseline was correlated with greater reductions in HbA1c at 3–12 months (*r* = −0.514, *p* < 0.001; Figure 1). Of the 304 participants, 124 (41%) experienced a > 5‐mmol/mol reduction in HbA1c at 3–12 months; this was likelier in younger individuals (58 [49–66] vs. 62 [54–70] years; *p* = 0.002) and those with higher HbA1c at baseline (84 [73–97] vs. 66 [55–76] mmol/mol; *p* < 0.001) but was not associated with sex (40% of women vs. 42% of men; *p* = 0.735), diabetes duration (17 [11–22] vs. 18 [12–24] years; *p* = 0.231), BMI (31.6 [28.0–35.1] vs. 31.5 [27.4–36.7] kg/m^2^; *p* = 0.900) or socioeconomic deprivation quintile (39% in the most deprived quintile vs. 42% in the least deprived quintile; *p* = 0.841). In logistic regression analysis, HbA1c response >5 mmol/mol (at 3–12 months) was only independently associated with HbA1c at baseline (odds ratio [OR] 1.06 per mmol/mol, 95% confidence interval [CI] 1.046–1.063; *p* < 0.001) but not with age (OR 0.994 per year, 95% CI 0.987–1.002; *p* = 0.135), BMI (OR 1.014 per kg/m^2^, 95% CI 0.995–1.036; *p* = 0.149) or diabetes type (OR for type 1 diabetes vs. type 2 diabetes 0.998, 95% CI 0.726–1.375; *p* = 0.989).

**TABLE 1:** Change in HbA1c at 3–12 months, 2 years and 3 years after initiation of Freestyle Libre use, stratified by baseline HbA1c category.

Baseline HbA1c category	Median (IQR) Δ HbA1c at 3–12 months, mmol/mol (median 271 days—*n* = 304)	*p* value	Median (IQR) Δ HbA1c at 2 years, mmol/mol (median 609 days—*n* = 226)	*p* value	Median (IQR) Δ HbA1c at 3 years, mmol/mol (median 963 days—*n* = 143)	*p* value
Total	−2 (−13 to 5)	<0.001	−2 (−15 to 7)	0.003	−2 (−16 to 9)	0.038
<58 mmol/mol	5 (−1 to 10)	<0.001	8 (4 to 13)	<0.001	6 (1 to 14)	0.003
58–75 mmol/mol	0 (−8 to 5)	0.619	0 (−9 to 13)	0.595	3 (−9 to 10)	0.511
>75 mmol/mol	−11 (−23 to 0)	<0.001	−14 (−26 to 0)	<0.001	−15 (−30 to −3)	<0.001

## 4 CONCLUSIONS

These data suggest that those with highest HbA1c at baseline have a clinically relevant reduction in HbA1c following commencement of Freestyle Libre use. The durability of these results, over a 3‐year period, suggest this is a real phenomenon rather than regression to the mean in people with high baseline HbA1c. Importantly, these changes are entirely consistent with the magnitude of change observed in type 1 diabetes. We were unable to collect data on hypoglycaemia and quality of life, which are other important clinical metrics potentially influenced by Freestyle Libre use.^1^ Indeed, the small rise in HbA1c in people with lower HbA1c at baseline may reflect therapy changes to avoid hypoglycaemia. We cannot exclude the possibility of HbA1c reduction caused by additional therapy changes, although these people were long established on basal‐bolus insulin regimens and the introduction of other glucose‐lowering agents, at this stage, is not typical. These data, when considered in the context of previously published data, support the use of Freestyle Libre in people with type 2 diabetes using basal‐bolus insulin regimens. It will be of interest to determine whether a wider role exists for Freestyle Libre in those on twice‐daily, basal‐only insulin regimens and in non‐insulin‐treated type 2 diabetes. To date, only relatively small studies have assessed these cohorts, suggesting an urgent need for larger, clinically representative randomized controlled trials.

Abbreviation: HbA1c, glycated haemoglobin; IQR, interquartile range.

### PEER REVIEW

The peer review history for this article is available at https://www.webofscience.com/api/gateway/wos/peer‐review/10.1111/dom.16003.

## Data Availability

The data that support the findings of this study are available on request from the corresponding author. The data are not publicly available due to privacy or ethical restrictions.
